# Acid–base Homeostasis and Implications to the Phenotypic Behaviors of Cancer

**DOI:** 10.1016/j.gpb.2022.06.003

**Published:** 2022-07-01

**Authors:** Yi Zhou, Wennan Chang, Xiaoyu Lu, Jin Wang, Chi Zhang, Ying Xu

**Affiliations:** 1 Cancer Systems Biology Center, China–Japan Union Hospital, Jilin University, Changchun 130033, China; 2 Department of Biochemistry and Molecular Biology and Institute of Bioinformatics, University of Georgia, Athens, GA 30602, USA; 3 Department of Medical and Molecular Genetics, Indiana University School of Medicine, Indianapolis, IN 46202, USA; 4 Department of Electrical and Computer Engineering, Purdue University, West Lafayette, IN 47907, USA; 5 Department of Biohealth Informatics, Indiana University–Purdue University Indianapolis, Indianapolis, IN 46202, USA; 6 Departments of Chemistry and of Physics and Astronomy, Stony Brook University, Stony Brook, NY 11794, USA

**Keywords:** Acid–base homeostasis, Cancer microenvironment, Metabolic reprogramming, Fenton reaction, Iron metabolism

## Abstract

**Acid–base homeostasis** is a fundamental property of living cells, and its persistent disruption in human cells can lead to a wide range of diseases. In this study, we conducted a computational modeling analysis of transcriptomic data of 4750 human tissue samples of 9 cancer types in The Cancer Genome Atlas (TCGA) database. Built on our previous study, we quantitatively estimated the average production rate of OH^−^ by cytosolic **Fenton reactions**, which continuously disrupt the intracellular pH (pH_i_) homeostasis. Our predictions indicate that all or at least a subset of 43 reprogrammed metabolisms (RMs) are induced to produce net protons (H^+^) at comparable rates of Fenton reactions to keep the pH_i_ stable. We then discovered that a number of well-known phenotypes of cancers, including increased growth rate, metastasis rate, and local immune cell composition, can be naturally explained in terms of the Fenton reaction level and the induced RMs. This study strongly suggests the possibility to have a unified framework for studies of cancer-inducing stressors, adaptive **metabolic reprogramming**, and cancerous behaviors. In addition, strong evidence is provided to demonstrate that a popular view that Na^+^/H^+^ exchangers along with lactic acid exporters and carbonic anhydrases are responsible for the intracellular alkalization and extracellular acidification in cancer may not be justified.

## Introduction

Acid–base homeostasis is a most fundamental property that all living cells must maintain, as the pH sets the stage for performing accurately all the biochemistry needed in support of the livelihood and the functionalities of the cells. pH is an essential part of probably all cellular processes of a living organism, which ensures the correct folding of proteins, biomolecular binding and interactions with the right affinity and specificity, conducting reactions at the desired rates in enzymatic pathways, among other biological functions. In normal human tissue cells, the intracellular (or cytosolic) pH, pH_i_, is generally neutral or slightly acidic at the range of 6.8–7.1, while the extracellular pH, pH_e_, is slightly basic at ∼ 7.2, with pH_i_ < pH_e_ [1]. Individual cellular compartments may have distinct pH levels required for executing their functions, with lysosomes having the most acidic pH at ∼ 4.0–5.0 and mitochondria having the highest pH at ∼ 8.0 [2]. Because of the vital importance of the pH, all living cells have designated systems to maintain the stability of their pH_i_ and pH_e_.

In human cells, a HP$O_{4}^{2-}$/$H_{2}$P$O_{4}^{-}$-based and a HC$O_{3}^{-}$/$H_{2}$C$O_{3}$-based buffering system are used to maintain the stability of pH_i_ and pH_e_, respectively, plus a suite of proton (H^+^) importers and exporters, such as Vacuolar-type Adenosine 5′-triphosphatase (V-ATPase), ${Na}^{+}/H^{+}$ exchanger, and ${Na}^{+}/HCO_{3}^{-}$ symporter. Under physiological conditions, certain metabolisms may produce large quantities of H^+^ such as *de novo* nucleotide biosynthesis [3] and the Warburg effect [4], while some other metabolisms may consume H^+^, such as the conversion of nicotinamide adenine dinucleotide from its reduced form (NADH) to its oxidized form (NAD^+^) [5]. Excess H^+^ or hydroxide (OH^−^) or equivalents produced dynamically by such acidifying or alkalizing processes are generally absorbed by the pH buffer and/or counterbalanced by H^+^ transporters to maintain the stability of the pH.

Persistent pathological conditions such as chronic inflammation are known to disrupt the stability of the local pH; and extracellular acidosis has been widely reported in diseased tissues across numerous human illnesses [6], [7], [8]. For example, cells in Alzheimer’s disease tissues are known to be under both extracellular and intracellular acidosis [9], [10]. Similar observations have been reported in other neurodegenerative diseases [11]. Diabetes is another example where the diseased tissue cells have been reported to have more acidic pH_e_ than the matching healthy tissues [12], [13]. Knowing the vital importance of the cellular pH stability, one could imagine the profound impacts of such changes on the whole cellular biochemistry. This is the reason that persistently altered pH has been suggested as a fundamental cause to a wide range of the altered metabolisms, hence considerable cellular behaviors in neurodegenerative diseases, diabetes, and cancer.

It is noteworthy that for pathological conditions giving rise to persistent overproduction of H^+^ or OH^−^, the pH buffer, along with the H^+^ transporters, has only a limited power in maintaining the pH stability. The reasons are two-fold: (1) each pH buffer has a fixed capacity, which could absorb only limited H^+^ (or OH^−^) [14], [15] persistently generated under pathological conditions; and (2) H^+^ transporters generally do not work in a sustained manner as such transporters fall into two types, electroneutral cotransporters/antiporters and electrogenic transporters. For electroneutral cotransporters/antiporters, H^+^ are released from or loaded into cells at the expense of extruding or absorbing another ion, *e.g.*, Na^+^ or Cl^−^. Hence their persistent utilization will disrupt the homeostasis of the other ion, making them not a sustainable solution. Similar can be said about a H^+^ importer or exporter (or equivalent), since it is electrogenic and its persistent utilization will violate the electroneutrality of the host cells, another fundamental property that cells must keep to remain viable [16].

Cancer is an intriguing case in terms of the altered pH_i_ and pH_e_ levels, as it has been well established that the pH_i_ of cancer tissue cells becomes basic (at ∼ 7.4 or 7.5), while their pH_e_ becomes acidic (ranging from 6.4 to 6.8) [1], hence a reversal of pH_i_ < pH_e_ compared to normal tissue cells. Multiple proposals have been made regarding the possible causes for the reversal of the pH_i_ and pH_e_ levels. These include (1) up-regulated H^+^ exporters such as V-ATPase, Na^+^/H^+^ exchangers, and lactic acid exporters in cancer [17], [18], [19]; (2) increased utilization of carbonic anhydrases that convert extracellular CO_2_ released by cancer cells to HC$O_{3}^{-}$ and H^+^ [20], [21]; and (3) hypoxia due to poor blood supply and “respiratory bursts” by innate immune cells [22]. These proposals have addressed the possible reasons for the extracellular acidosis in cancer tissues, which is probably needed by the local immune cells [6] but do not actually answer the question: what has made the pH_i_ alkaline, as we will demonstrate in this study.

V-ATPase is generally employed in the membrane of intracellular compartments and used to acidify compartments like endosome or lysosome [23], [24]. It is also used in plasma membrane for acidification of the extracellular space only in specialized cells such as osteoclasts and kidney cells. However, there have been no experimental data supporting the proposal that V-ATPase is localized in the plasma membrane of cancer tissue cells, to the best of our knowledge, except for studies reporting that the H^+^ pump is localized in the plasma membrane of certain metastasizing cancer cell lines [25], [26]. Na^+^/H^+^ exchangers are an interesting case, which are driven by both the gradients of Na^+^ and H^+^ with Na^+^-in being with the gradient and H^+^-out against the gradient when reversing pH_i_ and pH_e_. We will demonstrate here that the potential generated by Na^+^-in is insufficient to drive H^+^-out in cancer tissues. Lactic acid [C$H_{3}$CH(OH)C$O_{2}^{-}$ + H^+^] exporters like MCT1 are used by possibly all cancers in a sustained manner, as long as the acid is continuously produced by cancer cells [27]. We will demonstrate that for cells relying on the Warburg effect for adenosine triphosphate (ATP) production, lactic acid exporters do not contribute to intracellular alkalization.

Overall, the existing proposals did not adequately answer the question about the observed reversal of the pH_i_ and pH_e_ levels. Hence, further studies are needed. We have previously proposed a fundamentally different reason for the considerable alkalization of the pH_i_ in cancer tissue cells for most, possibly all cancer types [28].

**Supplementary Figure S1:**
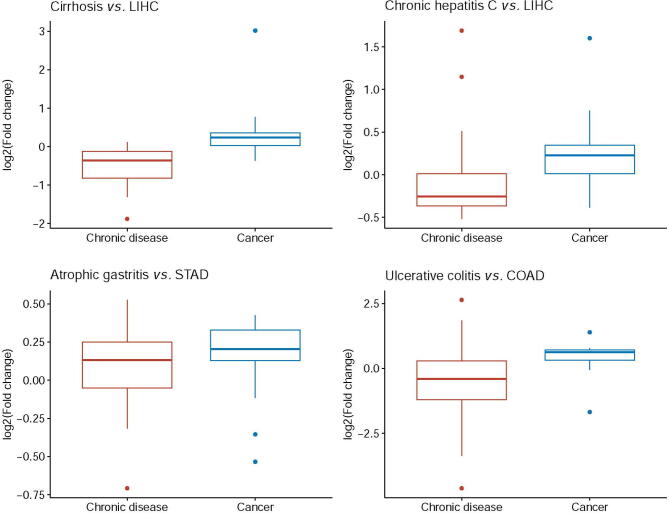
**Fenton reaction levels in cancer and non-cancerous chronic disease tissues**. The Y-axis shows the log2(fold change) of 20S proteasome genes in disease vs. control tissues. RNA-Seq data of chronic disease tissues were retrieved from GSE123661, GSE163416, GSE159008, and GSE84346. Differential expression analysis was performed using DESeq2.

Chronic inflammation is known to be causally linked to cancer onset and development [29], giving rise to increased local concentrations of H_2_O_2_. Once the H_2_O_2_ concentrations reach beyond a certain level, local red blood cells may become senescent due to the oxidation of their plasma membranes and their lack of a membrane repair mechanism [30], leading to their engulfment by macrophages [30] and local accumulation of irons released by macrophages after engulfment [31]. Under the condition of immune responses, local epithelial cells will sequester the free irons [32], leading to an overload of intracellular irons. It has been widely reported that multiple chronic inflammatory diseases [33] and many, possibly all cancer tissues have iron overload [34]. It is noteworthy that when both the H_2_O_2_ and Fe^2+^ levels are sufficiently high, Fenton reaction: Fe^2+^ + H_2_O_2_ → Fe^3+^ + ∙OH + OH^−^, an inorganic reaction without involving any enzymes, will happen [35], regardless being cancer or non-cancerous tissues. Fenton reactions have been widely observed in cancer tissues [36], [37], [38], and their levels are generally considerably higher in cancer compared with related non-cancerous disease tissues, as shown in Figure S1. Our previous work has discovered that all cancer tissue cells have Fenton reactions in their cytosol, mitochondria, extracellular matrix, and cell surface, respectively, and the reactions will continue if there are reducing molecules nearby that can convert Fe^3+^ back to Fe^2+^ such as superoxide ($O_{2}^{∙-})$, NADH, or vitamin C [28]. In addition, all cancers use $O_{2}^{∙-}$, generated by local immune cells and mitochondria of the cancer cells, as the main reducing molecules, which drives the cytosolic Fenton reactions continuously in the following form: $O_{2}^{∙-}$ + H_2_O_2_ → ∙OH + OH^−^ + O_2_, referred to as the Haber–Weiss reaction with Fe^2+^ serving as a catalyst [39], [40]. Furthermore, the rates of the cytosolic Fenton reactions in cancer can quickly saturate the pH_i_ buffer, hence driving the cytosolic pH up if the persistently produced OH^−^ is not neutralized. It is noteworthy to emphasize that persistent intracellular Fenton reaction is the result of chronic inflammation coupled with local iron overload, without involving any enzymes. One reliable way for computationally estimating the level of (cytosolic) Fenton reaction is through checking the level of 20S proteasome genes. The 20S proteasome is solely responsible for degradating protein aggregates formed due to interaction between hydroxyl radical (∙OH) and proteins, where ∙OH can only be produced intracellularly by Fenton reaction [28].

Now the question is: how do such Fenton reaction-affected cells keep their pH_i_ within a viable range? We have previously proposed a model regarding how cancer tissue cells reprogram numerous metabolisms [termed as reprogrammed metabolisms(RMs)] to produce H^+^ together at rates comparable to those of the cytosolic Fenton reactions, hence keeping their pH_i_ stable. The model is strongly supported by the observation that each of these RMs is found to produce more H^+^ or consume fewer H^+^ than its original metabolism [41]. The key RMs include (1) *de novo* biosynthesis of nucleotides; (2) the Warburg effect for ATP production; (3) simultaneous biosynthesis and degradation of triglyceride; and (4) overproduction and deployment of sialic acids and gangliosides. Here, we present a computational modeling analysis to provide further evidence that the RMs in each cancer tissue are indeed induced to produce H^+^ collectively at a rate comparable to the average rate of the cytosolic Fenton reaction. We also demonstrate that phenotypes known to be associated with specific cancer (sub)types can be naturally explained in terms of the induced RMs.

## Results

In this study, we conducted a modeling analysis to estimate quantitatively the level of cytosolic Fenton reaction in each cancer tissue and a regression analysis to predict which RMs are induced to produce H^+^ to keep the pH_i_ stable in the samples, followed by an association analysis between the known phenotypes or specific (sub)types and selected RMs in the relevant tissue samples. Overall, we applied our analyses to 4750 cancer samples, along with 503 matching control samples, across 9 cancer types (11 subtypes). All the samples were based on the transcriptomic data from The Cancer Genome Atlas (TCGA) database, and the single-cell RNA sequencing (scRNA-seq) data of head and neck squamous cell carcinoma (HNSC) and melanoma were used to validate our results.

### pH reversal by transporters?

Multiple proposals have been made regarding the possible causes of intracellular alkalization and extracellular acidification in cancer. One popular model is that Na^+^/H^+^ exchangers, particularly NHE1 (SLC9A1), are the main reason for the reversal of pH_i_ and pH_e_ in cancer tissues, along with monocarboxylate-H^+^ efflux cotransporters MCT1 (SLC16A1) and MCT4 (SLC16A3) and carbonic anhydrases for CO_2_ hydration [1]. Here we demonstrate that this possibility is low.

We found that among the nine cancer types under study, SLC9A1 was up-regulated in only three [breast invasive carcinoma (BRCA), HNSC, and thyroid carcinoma (THCA)] types, and down-regulated in six other types, as detailed in Table S1, indicating that SLC9A1 may not play a key role in most of the cancer types.

While ATP is known to be involved in the activation of SLC9A1, ATP is not involved in driving the transport [42]. Hence, the transporter is driven solely by gradients. Notably, the reversal of pH_i_ and pH_e_ requires the transporter to move intracellular H^+^ against the gradient out of the cell, indicating that the action must be driven by the gradient between the extracellular and intracellular sodium concentrations. It is known that the normal intracellular sodium concentration (ISC) ranges from 10 to 15 mmol/l, hence 12 mmol/l being used here, and the extracellular sodium concentration (ESC) is 140 mmol/l [43]. The total sodium concentration (TSC) in cancer (TSC_C_) is generally 2–3 folds of the matching normal one (TSC_N_) [44], [45]. The ratio between the extracellular and intracellular volumes in a unit tissue is 20:80 [46]. Our goal is to estimate the ISC in cancer (ISC_C_), assuming that the ESC (equivalent to blood sodium concentration) remains roughly stable. Hence, we have

(1) $${T\mathrm{SC}}_{C}=k\;{T\mathrm{SC}}_{N},\;with\;2\leq k\leq 3;\;and\;T\mathrm{SC}=0.2\times E\mathrm{SC}+0.8\times I\mathrm{SC}$$

By plugging the relevant numbers, we have

(2) $${T\mathrm{SC}}_{C}=0.2\times 140+0.8\times {I\mathrm{SC}}_{C};\;and\;{T\mathrm{SC}}_{N}=28+0.8\times {I\mathrm{SC}}_{N}=37.6$$

For *k* = 2, we have TSC_C_ = 28 + 0.8 × ISC_C_ = 2 × TSC_N_ = 75.2, and thus ISC_C_ = 47.2/0.8 = 59. For *k* = 3, we have ISL_C_ = 106. We conclude that the ISC_C_ should range from 59 to 106 mmol/l. Therefore, the ISC of a cancer tissue is on average 4.92–8.83 folds that of the matching normal tissue. Hence, the free energy gained for moving Na^+^ into the cells from the extracellular space for cancer cells can be calculated as follows:

(3) $$\Delta G_{{\mathrm{Na}}^{+}}\geq ZFV+RT\;ln\;\left(\frac{{{Na}^{+}}_{in}}{{{Na}^{+}}_{out}}\right)=1\times 96485.3\times \left(-0.07\right)+8.31\times 310\ln\left(\frac{59}{140}\right)$$

where *Z* = 1; *F* = 96,485.3 is the Faraday constant; *V* is the transmembrane potential (−0.07 meV inside membrane and 0 outside the membrane); *R* is the gas constant; and *T* is the temperature (room temperature). Similarly, the free energy needed for moving an intracellular H^+^ out of a cancer cell, using pH_e_ = 6.6 and pH_i_ = 7.4, can be calculated as follows:

(4) $$\Delta G_{H^{+}}=\;ZFV\;+\;RT\;ln\;\left(\frac{{H^{+}}_{out}}{{H^{+}}_{in}}\right)=1\times 96485.3\times \left(0.07\right)+8.31\times 310\ln\left(\frac{10^{-6.6}}{10^{-7.4}}\right)$$

Therefore, the total free energy for Na^+^-in and H^+^-out is $\Delta G_{{\mathrm{Na}}^{+}}+\Delta G_{H^{+}}$. Note that the first terms in the two free energies cancel each other, and the total free energy is:

(5) $$\Delta G_{{Na}^{+}}+\Delta G_{H^{+}}\geq 8.31\times 310\times \left(\ln\left(\frac{59}{140}\right)+\ln\left(\frac{10^{-6.6}}{10^{-7.4}}\right)\right)=8.31\times 310\times 0.978=2519\;J$$

The positive free energy indicates that the energy generated by Na^+^ import is insufficient to change the pH_e_ to 6.6 and the pH_i_ to 7.4, actually not even to pH_e_ = 6.8 and pH_i_ = 7.2 by SLC9A1. This calculation result is also experimentally supported [42]. It is noteworthy that the lower bound is used for the ISC_C_. Hence, a higher level of such concentration will make it more unlikely for the sodium gradient to drive the reversal of pH_e_ and pH_i_. Furthermore, the blood sodium concentrations in cancer patients are generally reduced, a widely known fact [47], suggesting that the actual $\Delta G_{{\mathrm{Na}}^{+}}$ + $\Delta G_{H^{+}}$ is higher than the value given in Equation (5).

Interestingly, the reversal of pH_e_ and pH_i_ is potentially achievable by SLC9A1 in normal tissue cells, where the ratio between intracellular and extracellular Na^+^ is 12:140, with

(6) $$\Delta G_{{Na}^{+}}+\Delta G_{H^{+}}=8.31\times 310\times \left(\ln\left(\frac{12}{140}\right)+\ln\left(\frac{10^{-6.6}}{10^{-7.4}}\right)\right)=8.31\times 310\times \left(-0.615\right)=-1583\;J$$

suggesting the possibility of reversing pH_e_ and pH_i_ by SLC9A1.

The conclusion here is that SLC9A1 could not accomplish the observed reversal of pH_e_ and pH_i_ because the Na^+^-in potential is considerably reduced in cancer due to the decreased ratio between intracellular and extracellular Na^+^ concentrations.

Lactic acid exporters SLC16A1 and SLC16A3 have also been suggested to play a role in intracellular alkalization in cancer. We can see from the following that this also is not true, when coupled with the Warburg effect. Note that ATP production by fermenting glucose is pH neutral as given below [48]:

(7) glucose + 2ADP^3−^ + 2HPO_4_^2−^ → 2 lactate^−^ + 2 ATP^4−^

which generates only a lactate but not lactic acid (lactate + H^+^) per ATP produced. Now, the question is where the H^+^ comes from when SLC16A1/SLC16A3 releases a lactic acid. Note that ATP hydrolysis (or consumption) produces one net H^+^ regardless of how the ATP is produced:

(8) ATP^4−^ + H_2_O → ADP^3−^ + HPO_4_^2−^ + H^+^

Hence, the Warburg effect coupled with ATP hydrolysis produces one net H^+^, which is co-released with the lactate. As a side note, ATP production by respiration consumes one H^+^ for each ATP produced:

(9) ADP^3−^ + HPO_4_^2−^ → ATP^4−^ + OH^−^

Hence, ATP production by respiration coupled with ATP hydrolysis is pH neutral. This is a fundamental difference between the two ATP production pathways.

We conclude that while SLC16A1/SLC16A3 contributes to the extracellular acidification, it does not contribute to the intracellular alkalization. Our previous work has provided strong evidence that cancer cells release the lactic acids mainly for modulating immune responses rather than pH homeostasis [49].

Carbonic anhydrases, particularly CA4 and CA7, have been suggested to play important roles in extracellular acidification in cancer. As shown in Table S2, *CA4* was either down-regulated or expressed at very low levels across all cancer types, except for stomach adenocarcinoma (STAD) where the expression slightly increased but remained at a very low level. Similarly, *CA7* was down-regulated or expressed at very low levels, except for THCA. These results indicate that the two genes do not play much roles in extracellular acidification in all nine cancer types.

### Estimation of OH^−^ production rates by cytosolic Fenton reactions

Our goal here is to construct a reliable metabolic network leading to cytosolic Fenton reaction and to estimate accurately the rate of the OH^−^ production by the Fenton reaction based on transcriptomic data of the available cancer tissues.

**Figure 1:**
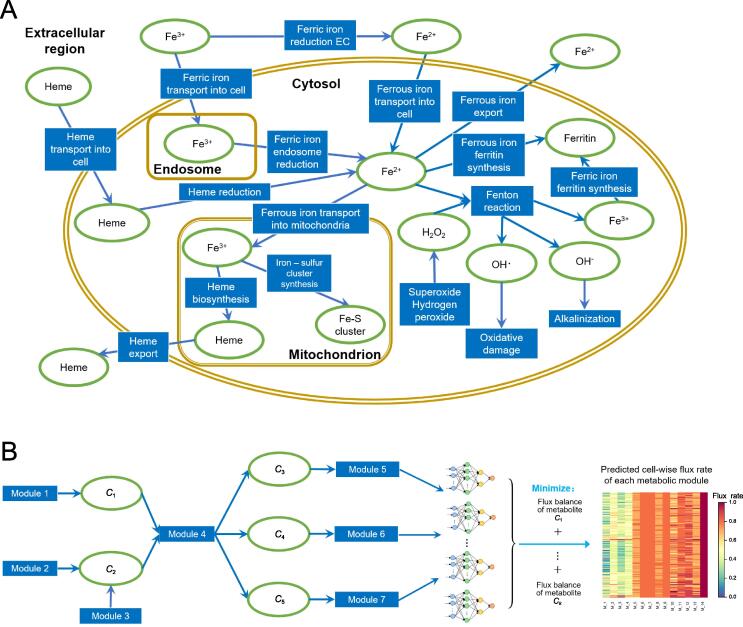
**Estimation of Fenton reactionlevels** **A.** A predicted map for iron metabolism relevant to cytosolic Fenton reaction in human cell. Reactions and metabolites are represented by blue rectangles and green ovals, respectively. **B.** Computational model of scFEA. Each metabolic reaction (or module) is modeled as a neural network of genes involved in the module. The parameters of the neural network were derived by minimizing the total flux imbalance of intermediate metabolites, an indicator for the quality of a predicted model. scFEA, single-cell flux estimation analysis.

To model the rate of the persistent cytosolic Fenton reaction: $O_{2}^{∙-}$ + H_2_O_2_ → ∙OH + OH^−^ + O_2_ (with Fe^2+^ as the catalyst), we need to estimate the concentration of each of the three reactants: H_2_O_2_, $O_{2}^{∙-}$, and Fe^2+^, and how each product is related to the reactants. Figure 1A depicts our constructed map of iron metabolic reactions in a human cell, which consists of three sources to the cytosolic Fe^2+^ pool, namely ferrous ion import, ferric ion import and reduction, and heme import and reduction; four sinks for the cytosolic Fe^2+^, namely mitochondrial Fe-S cluster, heme synthesis, ferrous ion export, and Fenton reaction; and sources and sinks of the $O_{2}^{∙-}$ and H_2_O_2_, totaling eight. The 15 metabolic branches were each considered as a metabolic module, each containing one to a few dozen of metabolic genes, whose expression levels were utilized to estimate their metabolic flux. Detailed information about gene names and rationale is given in Table S3.

We have recently developed a graph neural network-based method for predicting sample-specific metabolic rate, named single-cell flux estimation analysis (scFEA) [50]. Specifically, scFEA models metabolic fluxes in each tissue based on gene expression data of a large number of tissue samples, under two simple and reasonable assumptions: (1) the total influx of each metabolite is approximately the same as its total outflux; and (2) changes in the outflux of each metabolite can be modeled as a (non-linear) function of changes in the expression levels of genes involved in producing the metabolite. Note that assumption (1) is generally true unless some major in-/out-flux for a metabolite is not considered. Assumption (2) is a combination of two simpler assumptions: (i) the concentration of an enzyme is an (invertible) function of the concentrations of its reactants; and (ii) this concentration is also a (non-linear) function of the expression level of its encoding gene, with both functions being invariant across different samples of different cancer types. Both assumptions (1) and (2) are supported by published studies [51], [52], [53]. Intuitively, one can think this model as an integrated Michaelis–Menten model, whose parameters are implicitly estimated using the large number of available gene expression data.

Figure 1B outlines the workflow with further details given in Materials and Methods. Specifically, based on the two assumptions, scFEA models the metabolic flux of each module by a three-layer fully connected neural network of genes involved in the module, which minimizes the total imbalance of the intermediate substrates across all tissue samples. For a network with *X* modules, there are 12X×(#genes) unknowns to be estimated with #genes being the number of genes encoded in each reaction representing module, which is generally a small integer; and there are K×N constraints, where K and N are the numbers of intermediate substrates and samples, respectively. Hence, a network like the one depicted in Figure 1A, a transcriptomic dataset of more than 2000 samples such as the TCGA pan-cancer (and two selected scRNA-seq) data, should enable reliable estimation of the unknowns.

**Supplementary Figure S2:**
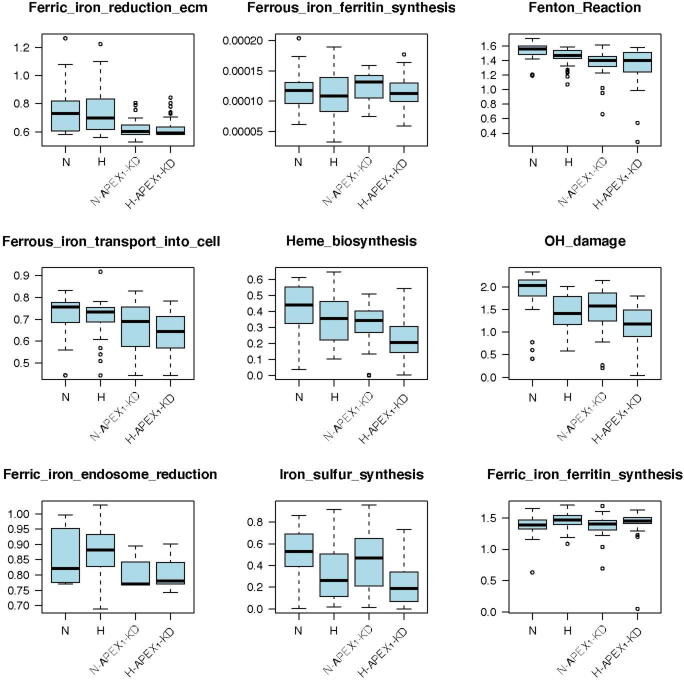
**Predicted flux of nine selected modules under four conditions**. The labels on the X-axis represent normoxic *APEX1* knockdown (N-*APEX1*-KD), normoxic control (N), hypoxic *APEX1* knockdown (H-*APEX1*-KD), and hypoxic control (H).

We have previously validated the scFEA algorithm on human global metabolic map and central metabolic pathways by using two sets of matched scRNA-seq and tissue metabolomic data [50]. Here, we further validated scFEA on the curated iron ion metabolic modules by applying the method on our recently collected scRNA-seq data of 168 patient-derived pancreatic cancer cell lines Pa03c under four conditions: normoxia (N), hypoxia (H), normoxia and knockdown of *APEX1* (N-*APEX1*-KD), and hypoxia and knockdown of *APEX1* (H-*APEX1*-KD). *APEX1* plays a central role in the cellular response to oxidative stress [54]. Our recent studies identified that knockdown of *APEX1* results in increased oxidative stress and cell death in Pa03c cells [55]. scFEA predicted that the levels of Fenton reaction, proteasome activity, and iron-sulfur cluster biosynthesis in normoxic cells were higher than those in hypoxic cells (Figure S2). These observations matched (1) the decreased levels of Fenton reactions under hypoxia condition due to the lack of oxygen and hydrogen peroxide, and (2) the decreased levels of tricarboxylic acid cycle-related iron-sulfur cluster-containing proteins. In addition, we observed that the level of ferric iron reduction was largely suppressed in *APEX1*-KD cells, as the overrepresented reactive oxygen species (ROS) might deplete ferrous iron pool in the cell (Figure S2).

**Supplementary Figure S3:**
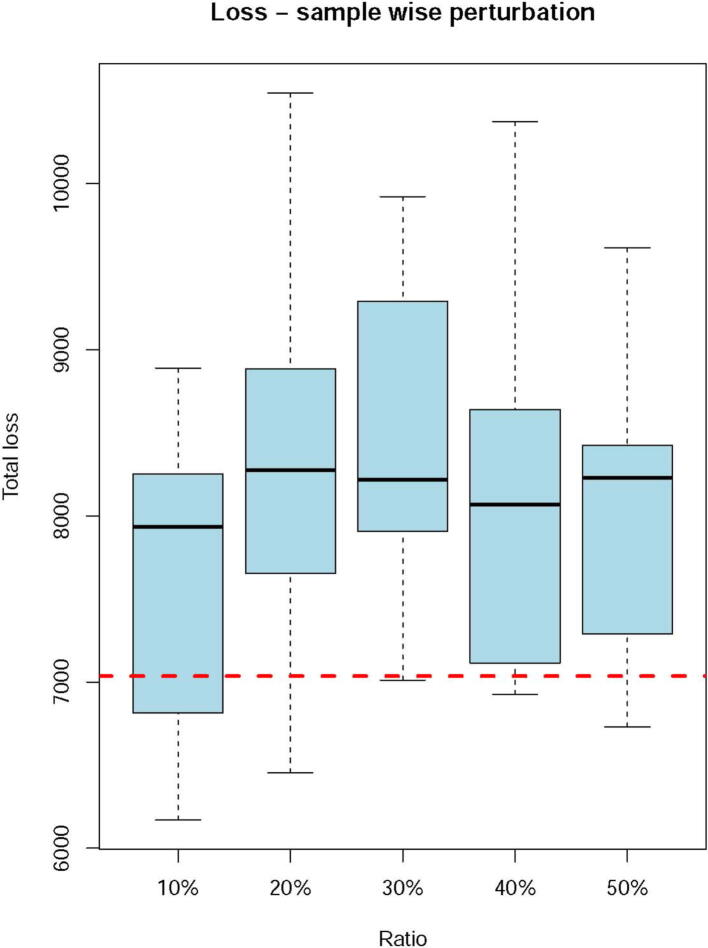
**Total loss of scFEA computed with different ratios of samples perturbed**. The X-axis shows the ratio of samples perturbed, with the baseline loss indicated as a red dashed line. The perturbation was conducted by first randomly selecting a certain proportion of samples and then randomly shuffling each row of the samples.

These observations demonstrated that the scFEA prediction can capture the major variations in iron ion metabolisms under different biochemical conditions. We also conducted a robustness analysis by using TCGA data. Similar to our past validation of scFEA [50], we randomly shuffled the gene expression profile of each iron ion metabolic genes in a certain proportion of samples and evaluated the total loss with respect to the level of perturbation. We observed higher total losses when perturbing more samples, which further demonstrated that the iron ion metabolic gene expression truly forms certain dependency over the curated metabolic modules (Figure S3).

### Iron metabolism in human cancer

**Figure 2:**
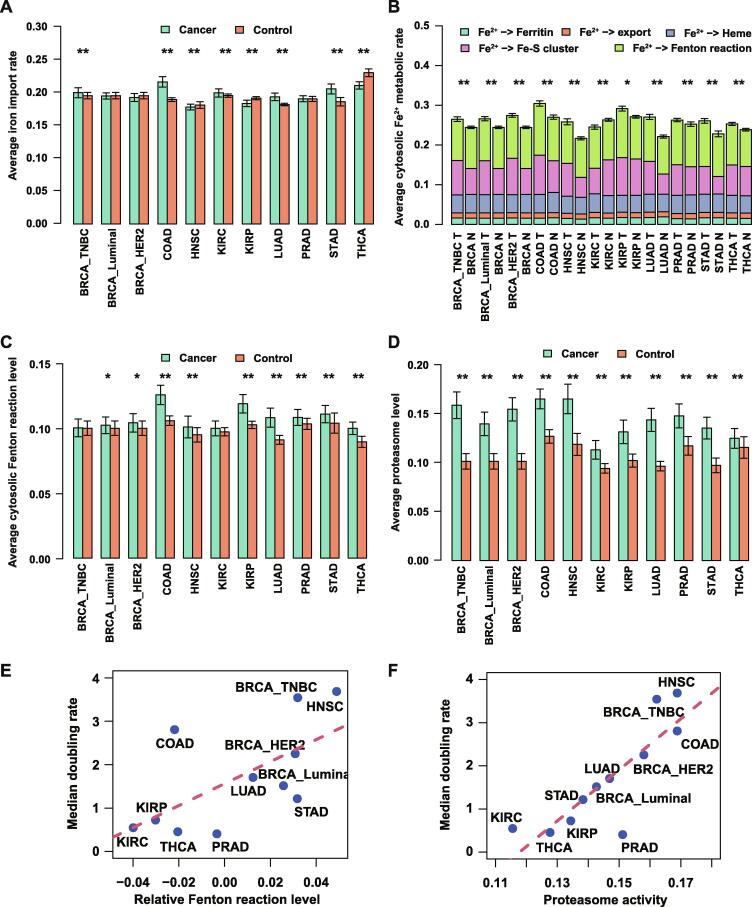
**Predicted iron fluxes** The predicted fluxes are relative flux levels scaled by a hyperparameter. **A.** Predicted average iron import rates (Y-axis) in cancer and adjacent control samples of each cancer type (X-axis). **B.** Predicted average cytosolic Fe^2+^ metabolic rates (Y-axis) by ferritin synthesis, mitochondrial heme synthesis, Fe-S cluster synthesis, Fe^2+^ export, and Fenton reaction, in cancer and adjacent control samples of each cancer type (X-axis). **C.** Predicted average cytosolic Fenton reaction levels (Y-axis) in cancer and adjacent control samples of each cancer type (X-axis). **D.** Predicted average proteasome levels (Y-axis) in cancer and adjacent control samples of each cancer type (X-axis). **E.** Correlation between the difference of relative Fenton reaction levels in cancer and control tissues (X-axis) and cancer growth rate (Y-axis). **F.** Correlation between the predicted proteasome activity level (X-axis) and cancer growth rate (Y-axis). *, *P* < 0.1; **, *P* < 0.05 (statistically significant difference). BRCA, breast invasive carcinoma; TNBC, triple-negative breast cancer; COAD, colon adenocarcinoma; HNSC,head and neck squamous cell carcinoma; KIRC, kidney renal clear cell carcinoma; KIRP, kidney renal papillary cell carcinoma; LUAD, lung adenocarcinoma; PRAD, prostate adenocarcinoma; STAD, stomach adenocarcinoma; THCA, thyroid carcinoma.

We first applied scFEA on all the samples of 9 cancer types and 11 subtypes (see Materials and Methods) compared with controls to predict the flux rates of the iron metabolism, as depicted in Figure 1A. Key prediction results are summarized in Figure 2.

Figure 2A summarizes the predicted uptake level of iron. Overall, five cancer (sub)types exhibited elevated iron uptake levels, including BRCA_TNBC (*P* = 0.043), colon adenocarcinoma (COAD; *P* = 2E−16), kidney renal clear cell carcinoma (KIRC; *P* = 0.02), lung adenocarcinoma (LUAD; *P* = 1E−10), and STAD (*P* = 1E−7); three showed reduced iron uptake levels, namely HNSC (*P* = 0.02), kidney renal papillary cell carcinoma (KIRP; *P* = 1E−5), and THCA (*P* = 3E−15); and three had approximately the same levels: BRCA_Luminal, BRCA_HER2, and prostate adenocarcinoma (PRAD). We also predicted the levels of five exits for cytosolic Fe^2+^, namely ferritin synthesis, heme, Fe-S cluster, Fe^2+^ export, and Fenton reaction, in cancers *vs*. controls. Overall, we observed that 10 out of the 11 cancer subtypes displayed higher cytosolic Fe^2+^ exit levels in cancer samples compared to their adjacent controls (*P* < 1E−3, except for KIRP), which is mostly due to the increased Fe-S biosynthesis and Fenton reaction, as detailed in Figure 2B. Furthermore, all 11 cancer subtypes displayed increased Fenton reaction levels compared with control tissues (Figure 2C), among which COAD (*P* = 6E−18), HNSC (*P* = 0.02), KIRP (*P* = 9E−12), LUAD (*P* = 8E−21), PRAD (*P* = 0.004), STAD (*P* = 0.01), and THCA (*P* = 9E−17) show significant changes, while BRCA_TNBC, BRCA_Luminal, BRCA_HER2, and KIRC exhibited slight but insignificant increases. On average, our prediction suggests that the Fenton reaction involves 39%–44% of the cytosolic Fe^2+^ utilization across the 11 cancer subtypes while more than 95% of the produced Fe^3+^ stored in ferritin. Statistics of all the iron ion metabolic modules in TCGA cancer types are provided in Table S4.

We also evaluated the level of the 20S proteasome which degrades particularly protein aggregates formed due to reaction with ∙OH [56] generated by Fenton reactions. Significantly increased proteasome levels were observed in all cancer types compared to controls (*P* < 1E−5, Figure 2D), providing an independent support for higher levels of cytosolic Fenton reactions in cancer tissues than in controls, knowing that ∙OH can only be produced intracellularly by Fenton reactions. The cytosolic Fenton reaction level could be related to the cell growth rate (Figure 2E and F), which is discussed in detail below.

### RMs induced for H^+^ production by alkalizing pH_i_

We have previously hypothesized that RMs observed in cancer tissue cells of the same cancer type are predominantly induced by cytosolic Fenton reactions to neutralize the OH^−^ produced by the reactions. The rationale for this hypothesis is a highly significant observation that two totally unrelated sets of reactions, namely cytosolic Fenton reactions and the total OH^−^ produced by the observed RMs are strongly statistically correlated [41]. In addition, multiple evidence strongly suggests that these two sets of reactions are causally linked and furthermore, Fenton reactions drive the induction of the RMs observed in each tissue sample but the other way around. Specifically, Fenton reaction is solely the result of increased innate immune responses, giving rise to increased H_2_O_2_ concentration and local iron overload and intracellular sequestration. In addition, none of the H^+^-producing RMs studied contribute to increased immunity or iron overload, based on our extensive literature review. Furthermore, OH^−^-producing Fenton reactions form a natural driver for the simultaneous induction of numerous RMs, which are distinct across different cancer types, to keep the pH_i_ stable. The other way around might require a biological program, which is orders of magnitude more complex than our current model. Based on these considerations, we tested our hypothesis using a more reliable way for estimating the quantities of the involved reaction rates.

A total of 43 RMs were analyzed with their names and marker genes listed in Table 1, including amino-acid biosynthesis and degradation, purine and pyrimidine biosynthesis, and lipid and fatty acid biosynthesis. For each RM, its level was assessed using single-sample Gene Set Enrichment Analysis (ssGSEA) on individual samples (see Materials and Methods) [57].

**Table 1:** **The 43RMs with names and marker genes**

**RM**	**Marker gene**
Arginine transportation	*SLC7A1*, *SLC7A2*, *SLC7A4*
Beta-oxidation	*ACAD10*, *ACAD9*, *ACADVL*, *CPT1A*, *ECH1*, *ECHS1*, *HSD17B10*, *EHHADH*
Ceramide synthesis	*SPTLC3*, *SPTLC1*, *SPTLC2*, *SPTSSA*, *KDSR*, *CERS1*, *DEGS1*, *SMPD1*, *SMPD2*, *SMPD4*, *SGMS1*, *SGMS2*, *SAMD8*
Choline production	*SLC44A1*, *SLC44A2*, *SLC44A3*, *CHKA*, *CHKB*, *PCYT1A*, *PCYT1B*, *CEPT1*, *CHPT1*, *AGMO*, *LYPLA1*, *LYPLA2*, *GDPD5*, *GPCPD1*, *HSD11B2*, *HSD17B2*, *AGPS*, *AGPAT1*, *LPIN1*, *PLA2G4A*
Chondroitin sulfate synthesis	*XYLT1*, *XYLT2*, *B4GALT7*, *B3GALT6*, *B3GAT1*, *B3GAT2*, *B3GAT3*, *CSGALNACT1*, *CSGALNACT2*, *CHSY3*, *CHPF*, *CHSY1*, *UST*, *CHST1*, *CHST2*, *CHST3*, *CHST7*, *CHST11*, *CHST12*, *CHST13*, *CHST15*
Circadian rhythm	*NPAS2*, *PER3*, *PER2*, *CSNK1D*, *CRY1*, *BHLHE41*, *BHLHE40*, *NR1D1*, *CRY2*, *CSNK1E*, *PER1*, *CLOCK*, *ARNTL*
Fatty acid synthesis	*FASN*, *MCAT*, *RPP14*, *RPP14*, *ACACA*, *ACACB*
Fatty acid transporter	*SLC27A1*, *SLC27A2*, *SLC27A3*, *SLC27A4*, *SLC27A5*, *SLC27A6*, *FABP1*, *FABP2*, *FABP3*, *FABP4*, *FABP5*, *FABP6*, *FABP7*, *FABP9*, *PMP2*
Gluconeogenesis-specific	*MDH1*, *MDH2*, *PC*, *PCK1*, *ENO1*, *ENO2*, *ENO3*, *BPGM*, *PGAM1*, *PGAM2*, *PGK1*, *PGK2*, *GAPDH*, *GAPDHS*, *ALDOA*, *ALDOB*, *ALDOC*, *FBP1*, *FBP2*, *GPI*, *G6PC*, *G6PC2*, *G6PC3*, *HK1*, *HK2*, *HK3*, *GCK*, *HKDC1*, *PFKL*, *PFKM*, *PFKP*, *TPI1*, *PKLR*, *PKM*
Glutaminolysis	*ME1*, *GOT2*, *GLS*, *SLC25A1*, *MDH2*, *ACLY*, *CS*, *SLC25A11*, *SLC25A13*, *OGDH*, *SDHA*, *SDHB*, *SDHC*, *SDHD*, *FH*
Heparan sulfate synthesis	*XYLT1*, *XYLT2*, *B4GALT7*, *B3GALT6*, *B3GAT1*, *B3GAT2*, *B3GAT3*, *EXTL2*, *EXTL3*, *A4GNT*, *EXT1*, *EXT2*, *EXTL1*, *HS2ST1*, *GLCE*, *NDST1*, *NDST2*, *NDST3*, *NDST4*, *HS3ST1*, *HS3ST3A1*, *HS3ST3B1*, *HS3ST2*, *HS3ST4*, *HS3ST5*, *HS3ST6*, *HS6ST1*, *HS6ST2*, *HS6ST3*
Hyaluronic acid synthesis	*PGM1*, *PGM2*, *UGP2*, *UGDH*, *GFPT1*, *GFPT2*, *GNPNAT1*, *PGM3*, *UAP1*, *HAS1*, *HAS2*, *HAS3*
Keratan sulfate synthesis	*CHST1*
Lysine degradation	*AASS*, *ALDH7A1*, *AADAT*, *DHTKD1*, *GCDH*, *ECHS1*, *HADH*, *HSD17B10*, *ACAT1*, *ACAT2*
Mevalonate pathway	*ACAT1*, *ACAT2*, *HMGCS1*, *HMGCS2*, *HMGCR*, *MVK*, *PMVK*, *MVD*, *IDI1*, *IDI2*, *GGPS1*, *FDPS*
*N*-glycosylation complex synthesis phase	*MGAT1*, *MAN2A1*, *MAN2A2*, *MGAT2*, *FUT8*, *MGAT3*, *MGAT4A*, *MGAT4B*, *MGAT4C*, *MGAT5*, *MGAT5B*, *B4GALT1*, *ST3GAL3*, *ST6GAL1*
*N*-glycosylation initial phase	*DPAGT1*, *ALG13*, *ALG1*, *ALG2*, *ALG11*, *ALG3*, *DPM1*, *DPM2*, *DPM3*, *ALG9*, *ALG12*, *ALG5*, *ALG6*, *ALG8*, *ALG10*, *OST4*, *STT3B*, *STT3A*
*N*-glycosylation processing phase	*MOGS*, *PRKCSH*, *GANAB*, *MANEA*, *MAN1C1*, *MAN1A2*, *MAN1B1*
*O*-glycosylation	*GALNT1*, *GALNT10*, *GALNT11*, *C1GALT1*, *ST3GAL1*, *ST3GAL2*, *GCNT1*, *GCNT3*, *GCNT4*, *GCNT7*, *B3GNT3*
Phospholipid degradation	*PLA2G6*, *PTGS1*, *PTGS2*, *PTGIS*, *PTGDS*, *HPGDS*, *PTGES*, *PTGES2*, *PTGES3*, *TBXAS1*, *ALOX5*, *LTA4H*, *LTC4S*, *GGT5*, *GGT3P*, *DPEP2*, *DPEP1*
Phospholipid synthesis-PA	*PGS1*, *PTPMT1*
Phospholipid synthesis-PC	*CHKA*, *CHKB*, *PCYT1A*, *PCYT1B*, *CEPT1*, *CHPT1*
Phospholipid synthesis-PE	*ETNK1*, *ETNK2*, *CHKB*, *PCYT2*, *CEPT1*, *EPT1*
Phospholipid synthesis-PI	*CDIPT*
Phospholipid synthesis-PS	*PTDSS1*
Proline synthesis	*ALDH18A1*, *PYCR1*, *G6PD*, *PGLS*, *PGD*, *PYCR2*
Purine dRN *de novo* synthesis	*PPAT*, *GART*, *PFAS*, *PAICS*, *ADSL*, *ATIC*, *IMPDH1*, *IMPDH2*, *GMPS*, *GUK1*, *NME4*, *NME1*, *NME2*, *NME7*, *RRM1*, *RRM2*, *RRM2B*, *ADSSL1*, *ADSS*, *AK5*, *AK8*, *AK3*
Purine dRN salvage synthesis	*PNP*, *APRT*, *ADK*, *ADA*, *HPRT1*, *IMPDH1*, *IMPDH2*, *GMPS*, *GUK1*, *RRM1*, *RRM2*, *RRM2B*, *NME1*, *NME2*, *NME4*, *NME7*, *ADSSL1*, *ADSS*, *ADSL*, *AK5*, *AK8*, *AK3*, *DGUOK*, *DCK*
Purine RN *de novo* synthesis	*PPAT*, *GART*, *PFAS*, *PAICS*, *ADSL*, *ATIC*, *IMPDH1*, *IMPDH2*, *GMPS*, *GUK1*, *NME4*, *NME1*, *NME2*, *NME7*, *RRM1*, *RRM2*, *RRM2B*, *ADSSL1*, *ADSS*
Purine RN degradation	*ADA*, *PNP*, *PGM2*, *RPE*, *RPIA*, *TKT*, *TALDO1*
Pyrimidine dRN *de novo* synthesis	*CAD*, *CMPK1*, *NME1*, *NME2*, *CTPS2*, *CTPS1*
Pyrimidine dRN salvage synthesis	*DCK*, *CDA*, *TK1*, *TK2*, *TYMS*, *RRM1*, *RRM2*, *RRM2B*, *NME4*, *NME1*, *NME2*, *NME7*, *DUT*, *ENPP3*, *ENPP1*, *ITPA*, *DTYMK*, *NTPCR*, *CANT1*
Pyrimidine RN *de novo* synthesis	*CAD*, *DHODH*, *UMPS*, *CMPK1*, *CMPK2*, *NME4*, *NME1*, *NME2*, *NME7*, *CTPS1*, *CTPS2*
Pyrimidine RN degradation	*CDA*, *UPP2*, *UPP1*, *DPYD*, *DPYS*, *UPB1*
Pyrimidine RN salvage synthesis	*CDA*, *UCK1*, *UCK2*, *UCKL1*, *CMPK1*, *CMPK2*, *NME4*, *NME1*, *NME2*, *NME7*, *CTPS1*, *CTPS2*
Reprogrammed lipid metabolism	*ACER1*, *ACER2*, *ACER3*, *CERS1*, *SGPP1*, *SGPL1*, *ACLY*, *FASN*, *SCD*, *FFAR1*, *FFAR2*, *FFAR3*, *FFAR4*, *GPR84*, *SLC27A3*, *HADH*, *HSD17B10*, *CPT1A*, *PPP1R14A*, *ACSL3*, *PCCB*, *DGAT1*, *DGKA*, *MOGAT1*, *LPCAT3*, *LIPE*, *PNLIP*, *LPL*, *DAGLA*, *DAGLB*, *CHKA*, *PTDSS1*, *CDIPT*, *SMPD2*, *SMPD1*, *SGMS1*, *SGMS2*, *S1PR1*, *S1PR2*, *S1PR3*, *PTGS1*, *PTGS2*, *ALOX5*, *TBXAS1*, *PTGIS*, *PTGES*, *PTGES2*, *PTGES3*, *PTGDS*, *LTA4H*, *LTC4S*, *GGT5*, *GGT3P*, *DPEP1*, *DPEP2*, *SPHK1*
Retinol biosynthesis	*RDH8*, *RDH10*, *RDH12*, *PNLIP*, *LIPC*, *RBP2*, *RBP1*, *RBP5*, *BCO1*
Retinol metabolism	*RDH16*, *SDR16C5*, *ALDH1A2*, *ALDH1A1*, *ALDH1A3*, *XDH*, *LRAT*, *CES1*, *CES2*, *RBP4*, *CES5A*, *CES4A*
Serine synthesis	*PHGDH*, *PSAT1*, *VPS29*, *PSPH*, *SLC1A4*, *SLC1A5*
Sialic acid synthesis	*GNE*, *NANS*, *NANP*, *CMAS*, *SLC35A1*
Triglyceride degradation	*LIPE*, *PNLIP*, *DAGLB*, *DAGLA*
Triglyceride synthesis	*GPAM*, *GPAT2*, *AGPAT1*, *MBOAT1*, *MBOAT7*, *MOGAT3*, *MOGAT1*, *DGAT2*, *DGAT1*, *LPCAT1*, *AGPAT6*, *LPPR3*, *LPPR4*
Tryptophan degradation	*TDO2*, *IDO1*, *IDO2*, *AFMID*, *KMO*, *KYNU*, *HAAO*, *ACMSD*, *ALDH8A1*, *DHTKD1*, *GCDH*, *ECHS1*, *HADH*, *HSD17B10*, *ACAT1*, *ACAT2*, *GOT2*, *AADAT*, *CCBL1*, *CCBL2*

*Note*: RM, reprogrammed metabolism; PA, phosphatidic acid; PC, phosphatidylcholine; PE, phosphatidylethanolamine; PI, phosphatidylinositol; PS, phosphatidylserine; dRN, deoxyribonucleotide; RN, pyrimidine ribonucleotide.

A linear regression analysis of the predicted levels of the cytosolic Fenton reactions against the levels of the 43 RMs was performed across all samples of the 9 cancer types (and 11 subtypes) with the L1 penalty for variable selection (see Materials and Methods). Table 2 shows the RMs, along with the numbers of H^+^ and CO_2_ produced, that are commonly and positively associated with Fenton reaction levels across all the samples, where the averaged contribution score and the rate of contribution represent the averaged regression parameter and the proportion of cancer types that the RMs were selected, respectively. Table S5 gives the selected RMs for each of the 11 cancer subtypes.

**Table 2:** **Contribution scores of RMs that are positively correlated with Fenton reaction levels**

**RM**	**Averaged contribution score**	**Rate of contribution**	**Net proton**
Purine dRN salvage synthesis	0.287	0.818	+1 per purine
Proline synthesis	0.23	1	+1 CO_2_ per proline
Tryptophan degradation	0.112	0.818	+1 per tryptophan
Pyrimidine RN salvage synthesis	0.094	0.727	+1 per pyrimidine
Pyrimidine dRN salvage synthesis	0.091	0.636	0 or +1 per pyrimidine
Phospholipid synthesis-PE	0.06	0.636	+1 CO_2_ per PE
Phospholipid synthesis-PA	0.054	0.818	+1 per PA
Phospholipid synthesis-PI	0.053	0.636	+1 per PI
Phospholipid synthesis-PS	0.052	0.455	+4 per PS
Phospholipid degradation	0.047	0.727	0 or +1 per phospholipid
Mevalonate pathway	0.041	0.545	+1 CO_2_ per farnesyl diphosphate
Gluconeogenesis-specific	0.041	0.545	+1 per pyruvate
Sialic acid synthesis	0.041	0.636	+2 per sialic acid
Fatty acid transporter	0.033	0.909	+1 per fatty acid
Beta-oxidation	0.032	0.455	+1 per fatty acid

*Note*: +*n* in column 4 denotes that *n* protons were produced.

Positive associations of the following RMs were identified in at least 40% of cancer types: purine deoxyribonucleotide (dRN) salvage synthesis, proline synthesis, tryptophan degradation, pyrimidine ribonucleotide (RN) salvage synthesis, pyrimidine dRN salvage synthesis, phospholipid synthesis, phospholipid degradation, mevalonate pathway, gluconeogenesis-specific, sialic acid synthesis, fatty acid transporter, and beta-oxidation, hence possibly representing the most commonly selected RMs in all cancers.

**Figure 3:**
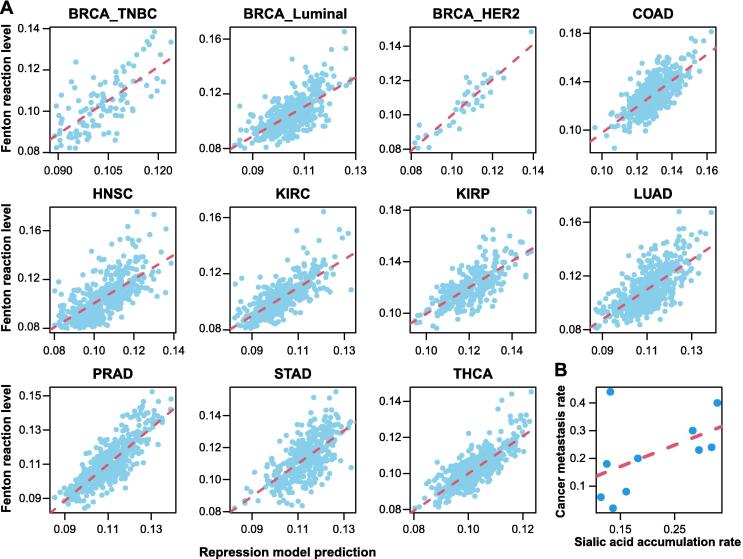
**A linear regression analysis of Fenton reaction levels *vs.* H^+^-producingRMs** **A.** Scatter plots showing the predicted Fenton reaction level (Y-axis) *vs*. the repression model prediction (X-axis) in 11 cancer subtypes. **B.** Scatter plot showing the known cancer metastasis rate (Y-axis) *vs*. the sialic acid accumulation rate. The sialic acid accumulation rate was a combined rate of sialic acid synthesis and degradation, and the sialic acid degradation rate was determined by the expression of the sialic acid degradation gene *NEU1*.

The selected RMs together achieved *R*^2^ > 0.8 in explaining the Fenton reaction level across all samples of the 11 cancer subtypes (Figure 3A). Specifically, purine, pyrimidine, and proline syntheses and tryptophan degradation showed the strongest associations with the predicted Fenton reaction levels and were commonly induced in more than 80% cancer types. It is noteworthy that nucleotide biosynthesis represents the most powerful acidifier, knowing that *de novo* synthesis of a purine produces 8–9 net H^+^ and that of pyrimidine produces 3–5 net H^+^ per nucleotide. Furthermore, proline synthesis is known to accelerate the glycolysis pathway [58] and also an effective producer of acids, as detailed below [41]:

(10) glutamate + ATP + G6P → proline + ADP + P_i_ + R5P + CO_2_

which produces one CO_2_ with per proline synthesized. Cancer generally utilizes a truncated tryptophan degradation pathway, whose end-product is kynurenine or 3-hydroxyanthrranliate rather than the usual acetyl-CoA for the full degradation pathway. There could be two possible reasons for the employment of the truncated pathway: (1) this process produces net H^+^; and (2) both end-products promote cell survival under immune attacks [59].

### Linking cancer phenotypes to Fenton reaction levels and induced RMs

Given that cellular phenotypes are dictated by the cell metabolisms, we further elucidated the possible relationships between the cancer phenotypes and the induced RMs.

#### *Cancer growth rate and cytosolic Fenton reaction level*

For each of the 11 cancer subtypes, we collected the average time needed for a tumor to double its volume, as detailed in Table 3. We observed a strong positive correlation, Pearson correlation coefficient (PCC) = 0.635 (*P* = 0.036) (Figure 2E), between the increase in the relative Fenton reaction levels in cancer tissues and adjacent control tissues, defined as the proportion of Fenton reaction among the total flux of the five cytosolic Fe^2+^ outfluxes (Figure 2B), and the tumor-growth rate, defined as $\frac{365}{days\;for\;tumor\;doubling}$. In addition, a stronger correlation was observed between the averaged 20S proteasome level and tumor growth rate, with PCC = 0.838 (*P* = 0.001) (Figure 2F). These findings provide strong evidence that the cytosolic Fenton reaction level plays a key role in determing the rate of cancer growth.

**Table 3:** **Average time needed to double the tumor size across 11 cancer subtypes**

**Cancer type**		**Median doubling time (day)**
BRCA		
TNBC		103
ER^+^		241
HER2		162
COAD		10
HNSC		99
KIRC		667
KIRP		504
LUAD		214
PRAD		900
STAD		300
THCA		803

*Note*: BRCA, breast invasive carcinoma; TNBC, triple-negative breast cancer; COAD, colon adenocarcinoma; HNSC, head and neck squamous cell carcinoma; KIRC, kidney renal clear cell carcinoma; KIRP, kidney renal papillary cell carcinoma; LUAD, lung adenocarcinoma; PRAD, prostate adenocarcinoma; STAD, stomach adenocarcinoma; THCA, thyroid carcinoma.

#### *Cancer metastasis and sialic acid accumulation*

Previous studies have suggested that the high level of sialic acid accumulation on cancer surface is associated with a high metastasis rate. We collected the metastasis rate of each cancer type under consideration and the synthesis of sialic acids. A positive correlation, PCC = 0.55 (*P* = 0.09), between the combined predicted rate of sialic acid synthesis and degradation and the metastasis rate of a cancer has been observed, providing strong evidence to the aforementioned speculation. In addition, we also conducted a regression analysis to fit the cancer type-specific metastasis rate against the sialic acid synthesis rate and the expression of the sialic acid degradation gene *NEU1*, giving rise to the following relationship:

(11) Metastasisrate=1.91×sialicacidsynthesis-0.039×NEU1

with *P* values of 0.071 and 0.076 for the two contributors, respectively. Hence, the analysis suggests a positive association between metastasis rate and sialic acids synthesis, and a negative association with sialic acid degradation, which together implies the rate of accumulation (Figure 3B).

#### *Local immune and stromal cell populations and Fenton reaction levels*

For each cancer type, we selected the 25% of the samples with the highest Fenton reaction levels, termed as samples with high Fenton reaction levels, and do the same on the 25% samples with the lowest Fenton reaction levels, termed as samples with low Fenton reaction levels. To study if the cytosolic Fenton reaction levels may be associated with certain immune and stromal cell types, we applied identification of cell types and deconvolution (ICTD), an in-house deconvolution method [60], to estimate the relative proportion of immune and stromal cells of different types in cancer samples of the nine cancer types. Our previous analysis demonstrated that ICTD could robustly identify 21 immune and stromal cell types and estimate their relative proportions by each cell type in TCGA samples (see Materials and Methods).

**Figure 4:**
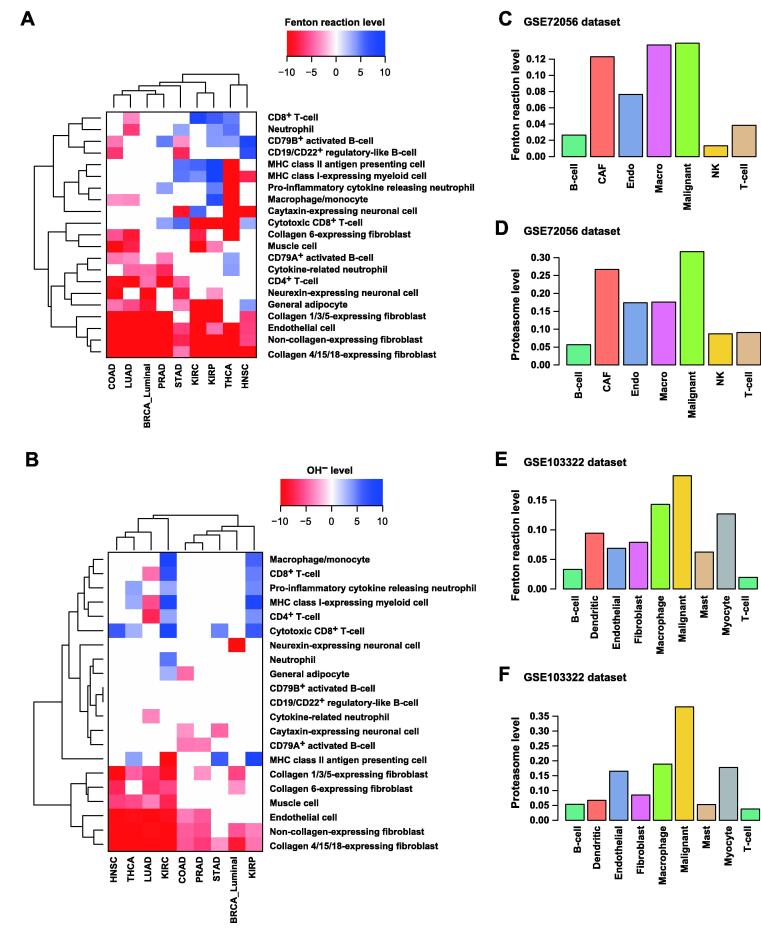
**Variations of tumor microenvironments associated with Fenton reaction and OH^−^levels** **A.** Differences in immune and stromal cell populations between samples of high and low Fenton reaction levels. **B.** Differences in immune and stromal cell populations between samples of high and low OH^−^ levels. **C.** and **D.** Predicted Fenton reaction levels (C) and proteasome levels (D) in each cell type in the GSE72056 dataset. **E.** and **F.** Predicted Fenton reaction levels (E) and proteasome levels (F) in each cell type in the GSE103322 dataset. Both Fenton reaction and proteasome levels were predicted by the relative flux rates. CAF, cancer-associated fibroblast; NK, natural killer.

In all the nine cancer types, the samples with high Fenton reaction levels tend to have fewer stromal cells, namely fibroblast cells, endothelial cells, muscle cells, adipocytes, and neural cells (Figure 4A). And such samples were negatively associated with the CD4^+^ T-cells and cytokine releasing neutrophils, all compared to the samples with low Fenton reaction levels. Furthermore, samples with high Fenton reaction levels exhibited higher proportions of major histocompatibility complex (MHC) class I/II-expressing cells, total T-cells, total B-cells, granulocytes, and cytotoxic CD8^+^ T-cells (Figure 4A).

We also compared the immune and stromal cell populations between the cancer samples with high and low OH^−^ levels (Figure 4B). We observed that cancer samples with high OH^−^ levels were negatively associated with stromal cell populations and positively associated with MHC class II antigen presenting cells, total T-cells, and total B-cells, especially the cytotoxic CD8^+^ T-cells.

**Supplementary Figure S4:**
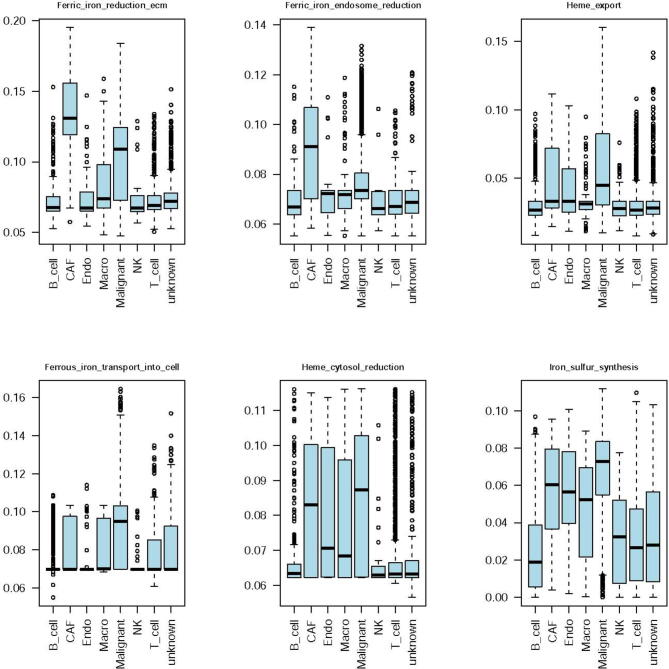
**Predicted flux of six selected modules in human melanoma**. The X-axis shows the eight cell types from the GSE72056 human melanoma scRNA-seq dataset.

**Supplementary Figure S5:**
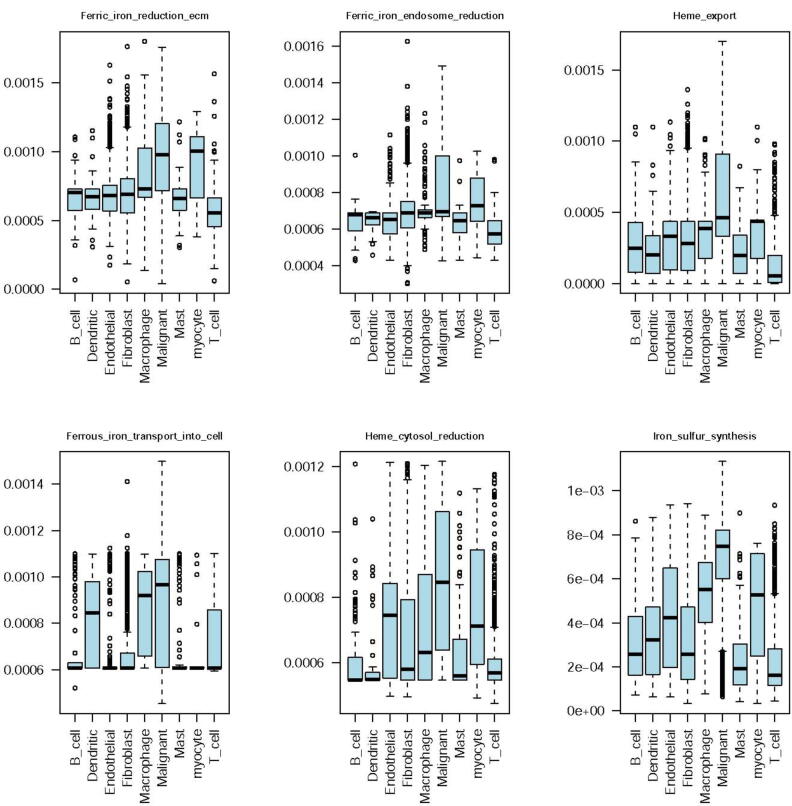
**Predicted flux of six selected modules in human head and neck cancer**. The X-axis shows the nine cell types from the GSE103322 human head and neck cancer scRNA-seq dataset.

We further conducted similar analyses on scRNA-seq data of human melanoma and HNSC. The results showed that in both cancer types, cancer cells had the highest Fenton reaction and proteasome levels (Figure 4C–F) among all cell types, which confirmed the aforementioned bulk data-based predictions using the TCGA samples. Other cell type-specific iron metabolic fluxes of these two datasets are provided in Figures S4 and S5.

## Discussion

Acid–base homeostasis and its persistent disruption are known to play key roles in, possibly at the roots of, the development of a wide range of chronic illnesses, ranging from type II diabetes, Alzheimer’s disease, and Parkinson’s disease [9], [11], [12], [13] to cancer. Here, we provide strong evidence that such disruption of the pH_i_, resulting from chronic inflammation and local iron accumulation, plays a driving role in the induction of a range of RMs for cell survival. Based on our analysis results, each cancer (sub)type utilizes a unique combination of RMs at specific levels (Table S5), together serving as a stabilizer of the disrupted pH_i_. Our modeling results strongly suggest that these induced RMs have given rise to the distinct phenotype of each cancer (sub)type. This, for the first time, provides a unified and effective framework for studying all RMs and cancerous behaviors in a systematic manner.

While our analysis is largely correlation-based, it has strong causal implications, since Fenton reactions are the results of chronic inflammation, and such reactions precede a majority of the metabolic reprogramming in diseases (unpublished data). With this understanding, our framework can be considered as that Fenton reactions drive metabolic reprogramming, which determines the altered cellular behaviors. In this sense, we propose that Fenton reactions may dictate a specific cancerous behavior. Note that we have focused on cytosolic Fenton reactions in this study, while Fenton reactions in other subcellular locations, namely mitochondria, extracellular matrix, and cell surface, as we have previously suggested [28], may lead to some other cancerous behaviors, which we did not discuss here.

Our analyses demonstrate that all the nine cancer types select *de novo* nucleotide biosynthesis as one of the top acidifiers to keep the pH_i_ stable. We observe that most of the nine cancer types utilize biosynthesis and deployment of sialic acids and gangliosides as a key acidifier. In addition, lipid metabolism was another major acidifier in a few cancer types.

Our previous work has provided strong evidence that the rate of *de novo* biosynthesis of nucleotides may dictate the rate of cell proliferation [28], hence explaining why different cancer (sub)types may have distinct proliferation rates as shown in the Results section. In addition, the rate of sialic acid accumulation has strong implications to the rate of cancer metastasis [61], thus giving an explanation of why non-small cell lung cancer, KIRP, head and neck cancer, and BRCA_HER2+ tend to have higher metastasis rates compared with others. Furthermore, different compositions of immune and stromal cell types in the cancer tissues are associated with different levels of Fenton reactions. We anticipate that a variety of other phenotypes of a cancer could also be naturally explained using this framework, possibly once Fenton reactions in other subcellular locations being considered, such as the levels of resistance to different drugs, the possible secondary locations of metastasizing cancers, and the possibilities of development of cachexia.

It should be noted that this framework not only provides a capability for explaining why a cancer (sub)type has specific phenotypes in terms of the induced RMs, but also enables studies of the possible relationships among different phenotypic characteristics of a cancer such as growth *vs*. metastatic rates. For example, we have learned from the Results section that the relationship between the rates of cancer cell proliferation and metastasis could be strongly correlated with nucleotide biosynthesis and sialic acid synthesis, respectively, which tend to serve as the top and the dominant acidifiers in cancer. Hence, for a given level of OH^−^ production in a cancer, a relatively higher level of nucleotide biosynthesis may imply a lower level for the sialic acid synthesis, since they together are utilized as the key acidifiers. We expect that similar arguments can be made about the relationships among other top acidifiers for a given cancer type.

### Fenton reaction and ferroptosis

Both local iron overload and Fenton reaction have been long and widely observed across numerous cancer types [28]. A natural question is: do cancer tissue cells tend to undergo ferroptosis? We have noted that among the key enzymes, ACSL4, LPCAT3, and ALOX15, responsible for converting polyunsaturated fatty acids (PUFAs) to PUFA-OOH (the main source of lethal lipid peroxides whose production leads to ferroptosis), at least two out of the three enzymes were significantly down-regulated or unchanged in seven out of nine cancer types, except for STAD (with two enzymes up-regulated), as detailed in Table S6. Further analyses revealed that the levels of the cytosolic Fenton reactions showed negative Spearman correlations with the marker genes for cellular response to hydroperoxides in all cancer types except for KIRC, LUAD, and STAD, as detailed in Table S7.

We also analyzed the differential expression of marker genes in tissue samples at different stages (I–IV) based on the clinical data retrieved from TCGA. At least one of the three key markers for ferroptosis, *ACSL4*, *LPCAT3*, and *ALOX15*, was down-regulated in all cancer types and stages. On average, 44 out of the 66 Fenton reaction marker genes were up-regulated in all cancers and stages. These differentially expressed genes also displayed a trend: the genes become more up- or down-regulated in advanced stages than in early stages. Detailed statistics of the differential expression of Fenton reaction and ferroptosis related genes are given in Table S8.

These findings indicate that cytosolic Fenton reactions in general do not contribute to but possibly prevent the production of lethal lipid peroxides, which leads to ferroptosis. Knowing that cytosolic Fenton reactions generally take place in either labile iron pool or in some iron-containing proteins like heme in cancer and that ∙OH generally travels no more 1 nm [62], we speculate that the ∙OH produced by Fenton reactions may not reach lipids, say in the membrane in cancers, and may even take away some Fe^2+^ from taking part in lipid peroxidation as our statistics suggest.

## Perspective

A key realization from this study is that the links from chronic inflammation and iron overload to pH-related stress and then to induced RMs and further to phenotypical features of each cancer type may represent the backbone of the development of a cancer, while other changes such as genomic mutations and epigenomic alterations may predominantly serve as facilitators for realization of this evolutionary process, as some authors have suggested [63], including our own [41]. Compared to signaling and regulatory processes, metabolic events are considerably more stable, as shown by common RMs shared by multiple cancer types. An important implication is that the issue of “drug resistance” could be potentially avoided by focusing on metabolisms rather than signaling/regulatory processes, since the issue of “drug resistance” essentially reflects the redundance (or robustness) nature of the signaling or regulatory processes in human cells and organs, hence suggesting a possibly new direction of enzyme-centric cancer treatment, *i.e.*, to inhibit key enzymes that acidify the cancer intracellular space and thus kill the cells.

It is noteworthy that this study examines the acid–base homeostasis and RMs from a perspective of chemical balances. We did not touch on issues related to the signaling and regulatory processes that connect disrupted homeostasis and induction of certain RMs nor touch on the roles played by genomic mutations as well as epigenomic activities in these inductions and their downstream activities. These could represent as future research directions to provide further mechanistic information about the functional role played by signaling and regulatory molecules in the induction of acidifying metabolisms. A related issue is to elucidate the possible reasons why certain metabolisms are induced to reprogrammed in some cancer types but not in other types, hence possibly leading to deepened understanding of specific cancer types and specific cancerous behaviors.

## Materials and methods

### Data collection

#### TCGA transcriptomic data

The TCGA RNA-seq v2 Fragments Per Kilobase of transcript, per Million reads mapped (FPKM) data of the nine cancer types (11 subtypes) were retrieved from the Genomic Data Commons (GDC) data portal (https://portal.gdc.cancer.gov/) using TCGAbiolinks [64]. Table 4 lists the names of the cancer types along with the information of the numbers of cancer and control samples. FPKM values were converted to transcripts per million (TPM) values as the latter is more stable across samples. Clinical data were obtained in the extensible markup language (XML) format from GDC and parsed with an in-house script. GENCODE gene annotations used by the GDC data processing pipeline were downloaded directly from the GDC reference files webpage.

**Table 4:** **Tumor and normal sample sizes in each cancer type**

**Abbreviation**	**Cancer type**	**Tumor sample count**	**Normal sample count**
BRCA	Breast invasive carcinoma	1091	113
COAD	Colon adenocarcinoma	456	41
HNSC	Head and neck squamous cell carcinoma	500	44
KIRC	Kidney renal clear cell carcinoma	530	72
KIRP	Kidney renal papillary cell carcinoma	288	32
LUAD	Lung adenocarcinoma	513	59
PRAD	Prostate adenocarcinoma	495	52
STAD	Stomach adenocarcinoma	375	32
THCA	Thyroid carcinoma	502	58

#### scRNA-seq data

We collected two scRNA-seq datasets from the Gene Expression Omnibus (GEO) database (https://www.ncbi.nlm.nih.gov/geo/).

The GSE72056 dataset was collected from human melanoma tissues. The original paper provided cell classification and annotations including B-cells, cancer-associated fibroblast (CAF) cells, endothelial cells, macrophage cells, malignant cells, NK cells, T-cells, and unknown cells [65].

The GSE103322 dataset was collected from HNSC tissues. The original paper provided cell classification and annotations including B-cells, dendritic cells, endothelial cells, fibroblast cells, macrophage cells, malignant cells, mast cells, myocyte cells, and T-cells [66]. Notably, as indicated by the original work, malignant cells have high intertumoral heterogeneity.

Basic processing was conducted using Seurat (v3) [67] with default parameters to filter out cells with high expression levels of mitochondrial genes. The cell type label and sample information provided in the original work were directly utilized.

### Software and statistical methods

#### ssGSEA

We applied the ssGSEA2.0 R package to estimate the levels of the selected RMs on individual samples [57]. The enrichment score (ES) computed by ssGSEA was utilized to represent the level of each RM. The gene sets of the RMs were collected and annotated in our previous work [50].

#### scFEA

We applied our scFEA method on the TCGA and two scRNA-seq data against the iron metabolic map. While the details of the method are given in [50], we outline the key ideas of the algorithm.

The inputs to scFEA are a gene expression dataset and a factor graph-based representation of the metabolic map. Let $FG\left(C^{1\times K},{\mathrm{RM}}^{1\times M},E=\left\{E_{C\to R},E_{R\to C}\right\}\right)$ be a given factor graph, where $C^{1\times K}=\left\{C_{k},k=1,\cdots ,K\right\}$ is the set of K metabolites, ${\mathrm{RM}}^{1\times M}=\left\{R_{m},m=1,\cdots ,M\right\}$ is the set of M metabolic reactions (represented as a rectangle in Figure 1A), and $E_{C\to R}$ and $E_{R\to C}$ represent direct edges from reaction $R_{m}$ to metabolite $C_{k}$ and from metabolite $C_{k}$ to reaction $R_{m}$, respectively. For the k-th metabolite $C_{k}$, define the set of reactions consuming and producing $C_{k}$ as $F_{\mathrm{in}}^{C_{k}}=\left\{R_{m}\right|{(R}_{m}\to C_{k})\in E_{C\to R}\}$ and $F_{\mathrm{out}}^{C_{k}}=\left\{R_{m}\right|\left(C_{k}\to R_{m}\right)\in E_{R\to C}\}$, which is derived from the stoichiometric matrix of the given metabolic map. For an RNA-seq dataset with N cells, denote $\mathrm{Flu}x_{m,j}$ as the flux of the *m*-th reaction in cell *j* (j=1,⋯,N), and $F_{j}=\left\{Flux_{1,j},\cdots ,Flux_{M,j}\right\}$ as the whole set of the reaction fluxes. Denote $G^{m}=\left\{G_{1}^{m},\cdots ,G_{i_{m}}^{m}\right\}$ as the genes associated with the reactions in $R_{m}$, and $G_{j}^{m}=\left\{G_{i_{1},j}^{m},\cdots ,G_{i_{m},j}^{m}\right\}$ as their expression levels in sample j, where $i_{m}$ is for the number of genes in $R_{m}$.

We model $\mathrm{Flu}x_{m,j}=f_{\mathrm{nn}}^{m}\left(G_{j}^{m}|\theta_{m}\right)$ as a multi-layer fully connected neural network with input $G_{j}^{m}$, where $\theta_{m}$ represents the parameters of the neural network. Then $\theta_{m}$ and cell-wise flux $\mathrm{Flu}x_{m,j}$ can be solved by minimizing the following loss function L, where λ serves as a hyperparameter:

(12) $$L=\sum_{j=1}^{N}\sum_{k=1}^{K}{\left(\sum_{{m\in F}_{\mathrm{in}}^{C_{k}}}Flux_{m,j}-\sum_{{m^{\prime }\in F}_{\mathrm{out}}^{C_{k}}}Flux_{m^{\prime },j}\right)}^{2}+\lambda {\sum_{j=1}^{N}\left(\sum_{m=1}^{M}Flux_{m,j}-TA_{j}\right)}^{2}$$

where $TA_{j}$ is a surrogate for the total metabolic level of cell j, which is assigned to a constant or total expression of all the metabolic genes in j.

It is noteworthy that this formulation defines a new graph neural network architecture for flux estimation over a factor graph, where each variable is defined as a neural network of biological attributes, *i.e.*, the genes involved in each reaction; and information aggregation between adjacent variables is constrained by the imbalance between the in- and out-fluxes of each metabolite.

#### Regression analysis of Fenton reaction levels vs. RM levels

We conducted a linear regression analysis to fit the Fenton reaction level against the RM levels across all samples of each cancer type. The R package glmnet was utilized for the regression analysis [68]. An L1 penalty was utilized for variable selection. The hyperparameter λ was determined through cross validation. The RMs positively associated with the Fenton reaction level in at least 40% of the cancer types under study were summarized.

#### Samples with high and low Fenton reaction and OH^−^ levels

We extracted the top and bottom 25% samples in terms of their predicted Fenton reaction levels in each cancer type as cancer type-specific high and low Fenton reaction samples. Similarly, we did that in terms of the OH^−^ levels.

#### Deconvolution analysis

We utilized our in-house deconvolution method, ICTD, to estimate the relative proportions among 21 immune and stromal cell types in each TCGA sample [60].

#### Statistical test of differential analysis

The Mann–Whitney test was used for all differential analysis, including differential gene expression analysis and the differential analysis of predicted flux.

## Code availability

The code for performing the analyses in this study can be found at https://github.com/changwn and https://github.com/y1zhou.

## Competing interests

The authors have declared no competing interests.

## CRediT authorship contribution statement

**Yi Zhou:** Methodology, Software, Formal analysis, Visualization, Writing – original draft, Writing – review & editing. **Wennan Chang:** Methodology, Software, Formal analysis, Visualization, Writing – original draft. **Xiaoyu Lu:** Software, Data curation. **Jin Wang:** Methodology, Validation, Writing – review & editing. **Chi Zhang:** Methodology, Validation, Supervision, Writing – original draft, Writing – review & editing. **Ying Xu:** Conceptualization, Validation, Supervision, Writing – original draft, Writing – review & editing. All authors have read and approved the final manuscript.

## Supplementary material

Supplementary Table S1 **Average expression levels of *SLC9A1* across all samples in control and four stages of each of nine cancer types**

Supplementary Table S2 **Average expression levels of *CA4* and *CA7* in control and each stage in nine cancer types**

Supplementary Table S3 **Genes used to estimate the flux of the 15 metabolic modules**

Supplementary Table S4 ***P* values of the difference of each iron ion metabolic module between each (sub)cancer type vs. normal control sample**

Supplementary Table S5 **Selected reprogrammed metabolisms for each of the 11 cancer subtypes**

Supplementary Table S6 **Log_2_ fold change of three key enzyme genes involved ferroptosis**

Supplementary Table S7 **Correlation coefficients between Fenton reaction level and hydroperoxide marker genes**

Supplementary Table S8 **Log_2_ fold change of Fenton reaction and ferroptosis related genes**
